# Impact of Blood Type O on Mortality of Sepsis Patients: A Multicenter Retrospective Observational Study

**DOI:** 10.3390/diagnostics10100826

**Published:** 2020-10-15

**Authors:** Daisuke Hasegawa, Kazuki Nishida, Takahiro Kawaji, Yoshitaka Hara, Yasuyo Shimomura, Kazuhiro Moriyama, Daisuke Niimi, Naohide Kuriyama, Ayumi Shintani, Hidefumi Komura, Osamu Nishida

**Affiliations:** 1 Department of Anesthesiology and Critical Care Medicine, Fujita Health University School of Medicine, 1-98 Dengakugakubo, Kutsukake-cho, Toyoake, Aichi 470-1192, Japan; hasegawa.daisuke.0407@gmail.com (D.H.); medcompass@gmail.com (T.K.); harahara19810105@gmail.com (Y.H.); yasuyo@fujita-hu.ac.jp (Y.S.); kuriyama@fujita-hu.ac.jp (N.K.); komura@fujita-hu.ac.jp (H.K.); 2 Department of Biostatistics, Nagoya University Graduate School of Medicine, 65 Tsurumai-cho, Showa-ku, Nagoya, Aichi 466-8550, Japan; nishida@med.nagoya-u.ac.jp; 3 Laboratory for Immune Response and Regulatory Medicine, Fujita Health University School of Medicine, 1-98 Dengakugakubo, Kutsukake-cho, Toyoake, Aichi 470-1192, Japan; anbix55@gmail.com; 4 Department of Anesthesiology, Nishichita General Hospital, Nakanoike 3-1-1, Tokai, Aichi 477-8522, Japan; d.niimi0429@gmail.com; 5 Department of Medical Statistics, Osaka City University Graduate School of Medicine, 1-4-3 Asahi-machi, Abeno-ku, Osaka-City, Osaka 545-8585, Japan; ayumi.shintani@gmail.com

**Keywords:** ABO blood type, 90-day mortality, risk stratification, septic shock, Sequential (Sepsis-related) Organ Failure Assessment (SOFA)

## Abstract

ABO blood groups have been implicated as potential risk factors for various diseases. However, no study has investigated the association between sepsis mortality and ABO blood types. We aimed to evaluate the impact of these blood types on mortality in patients with sepsis and septic shock. This retrospective observational study was conducted at two general hospitals in Japan. Patients diagnosed with sepsis or septic shock were included and divided into four groups based on blood type (O, A, B, and AB). The association between type O vs. other types and 28- and 90-day mortalities was evaluated using multivariate logistic regression analysis adjusted for age, sex, and Sequential (Sepsis-related) Organ Failure Assessment score. This study included 415 patients, of whom 131 (31.6%), 171 (41.2%), 81 (19.5%), and 32 (7.7%) had type O, A, B, and AB, respectively. Blood type O was not associated with 28-day (odds ratio: 1.7 *p* = 0.08) or 90-day mortality (odds ratio: 1.53, *p* = 0.091). However, type O was significantly associated with higher 90-day mortality (odds ratio: 3.26, *p* = 0.009) in patients with septic shock. The role of ABO blood type in risk stratification for septic shock and the mechanisms that potentially affect the prognosis of sepsis patients need further investigation.

## 1. Introduction

Sepsis is defined as a life-threatening organ dysfunction induced by a dysregulated host reaction to infection [1], and it is reportedly the most common cause of death in hospitalized patients [2]. The incidence of sepsis increases annually, with more than 19 million people developing sepsis each year [3]. A clear and easy-to-use risk stratification model is important because sepsis patients need immediate therapeutic intervention to reduce morbidity and mortality.

The ABO blood types are determined by glycans that are displayed on the surfaces of erythrocytes and other cells [4]. The ABO blood typing is widely used in clinical practice, and associations between blood type and disease have been studied since the early 1900s [5,6,7,8]. Recent studies suggest that blood type O is a potential risk factor for cancer, myocardial infarction, acute kidney injury, venous thromboembolism, trauma, and gastrointestinal bleeding [9,10,11,12,13,14]. However, to the best of our knowledge, the prognostic value of ABO blood typing in sepsis has not been reported. 

Patients with blood type O have been reported to have 25–30% lower blood plasma levels of the von Willebrand factor (vWF) than those with non-O blood types [15,16]. Furthermore, it has been determined that neutrophil extracellular traps (NETs) [17], which are important for local defense against invading pathogens, remain anchored to the vascular wall via the vWF and have significant neutrophil elastase proteolytic activity [18]. Taken together, these factors suggest that a person’s blood type may affect the disease pathophysiology, particularly concerning sepsis and its association to coagulation and immunology. Therefore, we hypothesized that blood type O sepsis patients have a higher risk of death, and that this effect is stronger in the severe septic shock groups because they are characterized by a more severe coagulation dysfunction.

## 2. Materials and Methods

This retrospective observational study was conducted in the Fujita Health University Hospital and Nishichita General Hospital. Patients diagnosed with sepsis, who were admitted to the intensive care unit (ICU) between 1 January 2013 and 31 December 2017, were included (since 1 May 2015, for those of the Nishichita General Hospital because the ICU was established only in May). Inclusion criteria were as follows: patients 18 years old or older, admission to the ICU with suspicion of severe infection, and diagnosis of sepsis or septic shock according to the Sepsis-3 definition [1]. The exclusion criterion was unknown patient outcomes (i.e., alive or dead within 28 or 90 days). The patients were divided into four groups according to the blood type (i.e., O, A, B, and AB), and the outcome differences between those with blood type O and non-O blood types were further evaluated. This analytical method was chosen based on the a priori hypothesis that blood type O has a negative impact on mortality in patients with sepsis and septic shock. The following information was retrospectively collected from medical records: age, sex, height, body weight, source of infection, lactate value, blood type (O, A, B, or AB), Acute Physiology and Chronic Health Evaluation (APACHE) II score [19], Sequential (Sepsis-related) Organ Failure Assessment (SOFA) score [20], and 28-day and 90-day mortalities. 

The primary outcome measures were 28- and 90-day mortalities. Sepsis was identified as described in the introduction [1]. Organ dysfunction was defined as an acute change in the total SOFA score ≥2 points after onset of the infection. The baseline SOFA score was assumed to be zero in patients with no known previous organ dysfunction. Septic shock was identified as a subgroup of sepsis, in which the underlying circulatory and cellular/metabolic abnormalities were profound enough to increase mortality substantially. Patients with septic shock were defined based on a clinical construct of sepsis, with persistent hypotension requiring vasopressors to maintain a mean arterial pressure of ≥65 mmHg and having a serum lactate level >2 mmol/L (18 mg/dL) in the absence of hypovolemia [1].

In the univariate analysis, continuous variables were compared using the Mann–Whitney U Test, while categorical variables were compared using the Fisher’s exact test. A multivariate logistic regression analysis was used to assess primary outcomes after simultaneously controlling for potential confounders. Variables incorporated into the model were selected based on clinical perspective and included age, sex, and SOFA score. Using the chi-squared test, the ABO blood type distribution was assessed in the septic population and the septic shock group to determine if the distributions corresponded to the Japanese normal population. The numerical values in the text and tables represent the median (interquartile range), unless otherwise noted. All statistical analyses were performed using the R software version 3.4.3 and EZR (version 1.31; Saitama Medical Center, Jichi Medical University, Saitama, Japan), which is a graphical user interface for R (version 3.2.2, R Foundation for Statistical Computing, Vienna, Austria). A *p*-value of <0.05 was considered statistically significant [21].

This study was approved by the institutional review board of Fujita Health University and Nishichita General Hospital (Approval no.: HM18-190 and 30-25, respectively) (23 August 2018). The need for written informed consent was waived owing to the retrospective nature of this study.

## 3. Results

After excluding 76 patients according to the exclusion criterion, 415 patients with sepsis were included in this analysis. The study population was divided based on the ABO blood types: 131 patients with type O (31.6%); 171 with type A (41.2%); 81 with type B (19.5%); and 32 with type AB (7.7%).

The chi-squared test showed that the blood type distribution in the study cohort reflected the normal distribution (*p* = 0.438) within the Japanese population (O: A: B: AB = 3:4:2:1). The baseline characteristics of the patients and outcomes according to the blood type are shown in Table 1. Only age (*p* = 0.011) was statistically different among the four groups; all other characteristics were similarly distributed among the blood types. As patients with blood type O had the highest mortality rate, the differences in outcomes between those with blood type O and non-O blood types were further evaluated.

Table 2 compares the baseline characteristics between those patients with blood type O and all other blood types. Patients with blood type O were significantly younger than those with non-O blood types (median age: 68 years vs. 73 years, *p* = 0.001). Similarly, patients with blood type O had significantly higher mean SOFA scores than those with non-O blood types (8.0 vs. 7.0, *p* = 0.03). There was no significant difference in the APACHE II score [19] between patients with blood type O and those with non-O blood types.

The blood type distribution in the septic shock group was as follows: 40 patients with type O (34.5%); 52 with type A (44.8%); 16 with type B (13.8%); and 8 with type AB (6.9%), which also reflected the normal distribution within the Japanese population (*p* = 0.187). Table 3 shows the comparison of baseline characteristics between those with blood type O and all other blood types in the septic shock group.

Table 4 shows the adjusted odds ratios for blood type O in predicting 28- and 90-day mortalities in patients with sepsis and septic shock. After adjusting for age, sex, and SOFA score, blood type O was not associated with increased risk for 28-day (odds ratio: 1.7, *p* = 0.08) or 90-day mortality (odds ratio: 1.53, *p* = 0.091) in sepsis patients. However, in the septic shock group, blood type O was associated with higher 90-day mortality (odds ratio: 3.26, *p* = 0.009) but not with 28-day mortality (odds ratio: 1.65, *p* = 0.313).

## 4. Discussion

In this retrospective study, the association between blood type O and the 28- and 90-day mortalities was evaluated in 415 ICU patients with sepsis, 116 of whom had septic shock. The findings indicated that blood type O was associated with an increased risk of 90-day mortality in patients with septic shock. To the best of our knowledge, this is the first study to report the association between ABO blood types and mortality in patients with sepsis. Although the inherent risk from blood type O cannot be altered, adequate recognition of the risks allows for early identification of high-risk patients, potentially decreasing mortality in this group. Moreover, this finding may further increase our understanding of the pathophysiology of organ dysfunction induced by sepsis and septic shock [22,23] in relation to ABO antigens on the surface of various human cells and tissues. Patients with blood type O have 25–30% lower plasma levels of vWF than those with non-O blood types [15,16]. vWF plays a critical role in primary hemostasis after injury by mediating the adhesion of platelets to the subendothelium of damaged vessel walls and promoting the aggregation of activated platelets.

In addition, vWF plays another critical role through immunothrombosis. As one of the innate immune response systems and an important player in immunothrombosis released by neutrophils, NETs appear to play a role in early host defense against bacterial dissemination [17]. NETs capture bacteria by forming thrombi in local areas [24], remaining anchored to the vascular wall via vWF and encompassing significant neutrophil elastase proteolytic activity [18]. The role of NETs during infections has been previously reported in several studies [24,25,26,27,28,29]. It is also supported by an increased susceptibility to infections due to a deficiency in NET production or destruction of the NET scaffold by DNases [24,25,26,27,28,29]. Therefore, lower plasma vWF levels in blood type O could affect the NET activity, possibly affecting coagulation and the immune response related to sepsis. 

However, the differences in the mechanisms underlying NET formation among the blood types remain unknown. Further studies are warranted to determine the role of blood type in the formation of NETs and the effect on the human body, possibly leading to the identification of a new potential therapeutic target in patients with sepsis.

This study had some limitations. First, as this was a retrospective study with a limited sample size and some patients were excluded due to insufficient data on outcomes, the possibility of residual confounding factors and missing data bias could not be eliminated. Second, all the patients examined in this study were Asians; therefore, it is unclear whether the findings are applicable to other ethnic groups. Third, we were unable to collect information on patients’ comorbidities for an analysis of how they might have affected outcomes because data was unavailable for further analysis. Further studies, including large-scale prospective studies, are needed to obtain more definitive data.

## 5. Conclusions

Blood type O is associated with a higher risk of 90-day mortality in patients with septic shock. Further studies are needed to confirm the potential mechanisms through which blood type affects septic shock prognosis.

## Figures and Tables

**Table 1:** Baseline characteristics and outcomes according to blood type in the study cohort.

Blood Type	O	A	B	AB	*p*-Value
Total number/%	131/31.6%	171/41.2%	81/19.5%	32/7.7%	
Age, years	68 (59–75)	72 (63–78)	73 (65–80)	71.5 (64.75–82)	0.011
Men, n/%	90/68.7%	114/66.7%	55/67.9%	22/68.8%	0.984
Height, cm	161 (153.5–168)	160 (154–167)	161 (153–166)	161 (154–164)	0.829
BW, kg	53.2 (48.0–64)	55 (47.3–62.6)	50.6 (43.7–59.3)	53 (48–58)	0.074
BMI, kg/m^2^	21.3 (18.7–24)	21.3 (18.6–24)	20 (18–22.4)	20.8 (19.5–22.7)	0.083
Source of infection, n					
Abdominal	48	65	28	17	0.317
Intravascular	16	18	9	2	0.859
Central nervous system	0	3	1	0	0.527
Respiratory	43	57	28	6	0.396
Skin/joint	14	15	8	5	0.653
Urinary tract	4	9	4	1	0.825
Unknown	6	4	3	1	0.724
APACHE II score	23 (17.5–32)	24 (18–29)	22 (17–30)	24 (15.25–31.25)	0.731
SOFA score	8 (5–11)	7 (4–10)	7 (4–10)	7 (6–10.25)	0.141
Lactate, mmol/L	1.6 (1.1–3.3)	2 (1.1–3.7)	1.7 (1.1–2.9)	1.3 (0.9–2.7)	0.311
28-day mortality, n/%	23 (17.6)	25 (14.6)	7 (8.6)	5 (15.6)	0.352
90-day mortality, n/%	41 (31.3)	42 (24.6)	17 (21.0)	9 (28.1)	0.363

Data are presented as the median and interquartile ranges (25–75% percentile) or as absolute frequencies with percentages. Lactate values were measured on admission to the intensive care unit. BW, body weight; BMI, body mass index; APACHE, Acute Physiology and Chronic Health Evaluation; SOFA, Sequential (sepsis-related) Organ Failure Assessment.

**Table 2:** Comparison of baseline characteristics between patients with blood type O and other blood types among the study cohort.

Blood Type	O	Non-O	*p*-Value
Total number/%	131/31.6%	284/68.4%	
Age, years	68 (59–75)	73 (64.75–79.25)	0.001
Men, n/%	90/68.7%	191/67.3%	0.857
Height, cm	161 (153.5–168)	160 (154–166)	0.424
BW, kg	53.2 (48–64)	53 (46–60.75)	0.165
BMI, kg/m^2^	21.3 (18.7–24)	20.9 (18.4–23.4)	0.19
Source of infection, n			
Abdominal	48	110	0.745
Intravascular	16	29	0.611
Central nervous system	0	4	0.313
Respiratory	43	91	0.910
Skin/joint	14	28	0.861
Urinary tract	4	14	0.449
Unknown	6	8	0.386
APACHE II score	23 (17.5–32)	23 (17–30)	0.453
SOFA score	8 (5–11)	7 (4–10)	0.03
Lactate, mmol/L	1.6 (1.1–3.3)	1.8 (1.1–3.5)	0.746
28-day mortality, n/%	23/17.6%	37/13%	0.285
90-day mortality, n/%	41/31.3%	68/23.9%	0.144

Data are presented as the median and interquartile ranges (25–75% percentile) or as absolute frequencies with percentages. Lactate values were measured on admission to the intensive care unit. BW, body weight; BMI, body mass index; APACHE, Acute Physiology and Chronic Health Evaluation; SOFA, Sequential (sepsis-related) Organ Failure Assessment.

**Table 3:** Baseline characteristics of patients with blood type O and others in the septic shock group.

Blood Type	O	Non-O	*p*-Value
Total number/%	40/34.5%	76/65.5%	
Age, years	71.5 (63.25–75.25)	74 (67–80)	0.053
Men, n/%	27/67.5%	55/72.4%	0.739
Height, cm	158 (152–168)	160 (154–166)	0.386
BW, kg	52.1 (44.9–60)	54 (44.9–60.1)	0.961
BMI, kg/m^2^	20.8 (18.7–23.2)	20.6 (18.4–23.9)	0.672
Source of infection, n			
Abdominal	22	28	0.077
Intravascular	1	6	0.419
Central nervous system	0	0	-
Respiratory	9	26	0.210
Skin/joint	3	6	>0.999
Urinary tract	2	4	>0.999
Unknown	3	6	>0.999
APACHE II score	32.5 (21.75–39.25)	28 (21.75–37.25)	0.335
SOFA score	12 (8.75–14.25)	11 (9–13)	0.588
Lactate, mmol/L	4.7 (2.8–9.0)	3.8 (2.7–6.5)	0.329
28-day mortality, n/%	11/27.5%	14/18.4%	0.372
90-day mortality, n/%	21/52.5%	21/27.6%	0.014

Data are presented as the median and interquartile ranges (25–75% percentile) or as absolute frequencies with percentages. Lactate values were measured on admission to the intensive care unit. BW, body weight; BMI, body mass index; APACHE, Acute Physiology and Chronic Health Evaluation; SOFA, Sequential (sepsis-related) Organ Failure Assessment.

**Table 4:** Adjusted odds ratios for blood type O in predicting 28- and 90-day mortalities in sepsis and septic shock patients.

Parameter	Sepsis Patients		Septic Shock Patients	
	28-day Mortality	90-day Mortality	28-day Mortality	90-day Mortality
Odds ratio (95% CI)	1.7 (0.94–3.06)	1.53 (0.94–2.5)	1.65 (0.62–4.38)	3.26 (1.34–7.9)
*p*-value	0.08	0.091	0.313	0.009

The model was adjusted for age, sex, and Sequential (Sepsis-related) Organ Failure Assessment score. CI, confidence interval.

## Abbreviations

vWF	von Willebrand Factor
NETs	Neutrophil Extracellular Traps
ICU	Intensive Care Unit
APACHE	Acute Physiology and Chronic Health Evaluation
SOFA	Sequential (Sepsis-related) Organ Failure Assessment
